# Paeonol Disrupts the Integrity of *Aspergillus flavus* Cell Walls via Releasing Surface Proteins, Inhibiting the Biosynthesis of β-1,3-Glucan and Promoting the Degradation of Chitin, and an Identification of Cell Surface Proteins

**DOI:** 10.3390/foods10122951

**Published:** 2021-12-01

**Authors:** Qian Li, Ying Zhao, Yanli Xie

**Affiliations:** Henan Key Laboratory of Cereal and Oil Food Safety Inspection and Control, College of Food Science and Engineering, Henan University of Technology, Zhengzhou 450001, China; lq@haut.edu.cn (Q.L.); zy18838014010@163.com (Y.Z.)

**Keywords:** paeonol, *Aspergillus flavus*, cell walls

## Abstract

Paeonol can effectively inhibit *Aspergillus flavus* (*A. flavus*) via damaging cell walls. In this work, paeonol treatment remarkably destroyed both the outer amorphous layer and the inner fibrous layer of cell walls. Furthermore, FT-IR and XPS characterization showed that OH functional groups were altered and proteins in the outer layer were released. According to proteomic analysis, 605 proteins have been identified and annotated. The activities of β-1,3-glucan synthase and chitinase were prohibited and promoted, respectively, by paeonol treatment, however, the activities of β-1,3-glucanase and chitin synthase were not influenced. QRT-PCR results suggested that *FKSP*, *CHIIII*, and *CHIV* genes might be the antifungal targets of paeonol. In addition, paeonol can effectively restrain the pathogenicity of *A. flavus* on peanut butter. This study provided a new elucidation on the mode of action of paeonol against cell walls of *A. flavus*, facilitating the application of paeonol in the preservation of agricultural products.

## 1. Introduction

*Aspergillus flavus* (*A. flavus*), a common filamentous fungus, is prone to contaminate peanuts, corns, and other food crops. *A. flavus* can cause aspergillosis in immunocompromised humans and animals [1]. In addition, the secondary metabolite aflatoxins (AFs) are severely carcinogenic, teratogenic and mutagenic, of which AFB_1_ is the most toxic and has been classified as a category I carcinogen by the cancer research organization of the World Health Organization (WHO) [2]. According to a recent report, about 25% of crops have been contaminated due to *A. flavus* infection [3]. The related economic loss across the world has reached up to hundreds of millions of dollars per year [4]. Therefore, how to effectively control the contamination of *A. flavus* is of great significance in ensuring the safety of grains of agricultural products and guaranteeing the health of humans and livestock.

The fungal cell wall is composed of various compounds, such as polysaccharides, proteins, fats, and ions, and is essential in maintaining cell homeostasis and protecting cells [5,6]. As a unique and dynamic structure, fungal cell wall has been extensively studied and considered as an attractive antimicrobial target [6,7,8]. Although there has been no report on the detailed components of *A. flavus* cell walls, in our previous study, they are assumed to be organized into two layers [9], similar to *Aspergillus fumigatus* (*A. fumigatus*). The outer amorphous layer contains *N*-glycosylated proteins and the fibrous inner layer contains mainly glucans and chitin [10,11]. In *A. fumigatus* mycelia, one carbohydrate part of this glycoprotein identified in the outer layer is galactosaminogalactan (GAG) that plays important roles in the primary pathogenicity to the host [12,13,14]. In addition, depending on the strains and growing state, surface proteins of *A. flavus* are diverse, therefore, identifying these proteins should be considered beneficial in understanding the infection of *A. flavus* on grains, as well as controlling its contamination.

Besides the commercial antifungal agents on the market, several plant-derived natural compounds have been reported to be capable of destroying cell walls to different extents, such as cinnamaldehyde [15], 2-hydroxy-4-methoxybenzaldehyde (HMB) [16], and *o*-vanillin [9]. In our previous study, we have screened a new natural antifungal compound-paeonol, which exerts a strong inhibitory effect against *A. flavus* via destroying cell surface and reducing the content of β-1,3-glucan and chitin [17]. All these proofs are directed to the assumption that paeonol can be developed as a substitute for commercial chemical fungicides (e.g., echinocandins [18] and nikkomycin [19]) against cell walls of *A. flavus*. However, the detailed mode of action of paeonol against cell walls is yet to be demonstrated.

In this study, we tried to reveal the antifungal effect of paeonol against *A. flavus* cell walls in three aspects: first, the ultrastructural changes of cell walls were characterized by transmission electron microscopy (TEM). Subsequently, the functional groups and the elements in the outer surface of cell walls were examined by Fourier transform infrared spectroscopy (FT-IR) and X-ray photoelectron spectroscopy (XPS), respectively. Notably, proteins in the outer layer of cell walls were identified by proteomic analysis. To determine which enzymes and genes contribute to the metabolisms of β-1,3-glucan and chitin in the cell wall, the activities of enzymes participating in their biosynthesis and hydrolysis and/or degradation were quantified, and relative expressions of several key genes encoding these enzymes were also quantified by real-time quantitative reverse transcription PCR (QRT-PCR). Finally, the antifungal efficacy of paeonol on peanut butter was also evaluated.

## 2. Materials and Methods

### 2.1. Chemicals

Paeonol (purity: 99%, CAS: 552-41-0, Shanghai Aladdin Bio-Chem Technology Co. Ltd., Shanghai, China) was dissolved in ethanol absolute and stored at 4 °C in darkness. Chitin (CAS: 1398-61-4), N-acetyl-D-glucosamine (GlcNAc, CAS: 512-17-6), Uridine 5′-diphospho-N-acetylglucosamine disodium salt (UDP-GlcNAc, CAS: 91183-98-1) and Brij^®^ 35 (CAS: 9002-92-0) were purchased from Shanghai Aladdin Bio-Chem Technology Co., Ltd. (Shanghai, China). Trypsin (CAS: 9002-07-7) and 3,3,5′,5′-tetramethylbenzidine (TMB, CAS: 54827-17-7) were purchased from Sangon Biotech Co. Ltd. (Shanghai, China). Wheat germ agglutinin (WGA, L3892, and WGA-HRP, L9640) were purchased from Sigma-Aldrich Trading Co. Ltd. (Shanghai, China). Uridine 5′-diphosphoglucose disodium salt (UDP-Glc, CAS: 28053-08-9) was purchased from Shanghai Macklin Biochemical Co., Ltd. (Shanghai, China). GTP solution (R0461) was purchased from Thermo Fisher Scientific Co., Ltd. (Shanghai, China).

### 2.2. Fungal Strain and Culture Conditions

*A. flavus* strain (CGMCC 3.6304, China General Microbial Culture Collection Center) was cultured on sabouraud dextrose agar (SDA: 4% glucose, 1% peptone, and 2% agar) at 28 ± 2 °C as described previously [20]. Mycelia were cultured in sabouraud dextrose broth (SDB: 4% glucose and 1% peptone).

### 2.3. Transmission Electron Microscopy

TEM characterization was performed according to Li et al. [9] with slight modifications. One hundred microliters of *A. flavus* spore suspension (5 × 10^5^ /mL) were added into 20 mL SDB and cultured for 24 h. Then, paeonol dissolved in ethanol was added to the SDB to reach the final concentrations of 0 and MIC (0.625 mg/mL, previously tested in our lab [17]). After another 24 h culture, the fresh mycelia were collected and fixed with 2.5% (*v*/*v*) glutaraldehyde and 4% (*v*/*v*) paraformaldehyde in 0.1 M phosphate-buffered saline (PBS) at 4 °C for 4 h followed by a postfixation with 1% (*w*/*v*) osmium tetroxide at 4 °C for 2 h. Afterward, the samples were dehydrated with 25%, 50%, 75%, 85%, 95%, and 100% ethanol, successively. The samples were then embedded in Spurr’s resin (SPI Supplies, West Chester, PA, USA). Ultra-thin sections (approximately 50 nm) were obtained by an ultramicrotome (Leica EM-UC7, Leica Ltd., Weztlar, Germany). The sections were mounted on copper grids and stained with 2% saturated uranyl acetate and lead citrate each for 7 min. At last, the samples were visualized with a transmission electron microscope (JEM-1400, JEOL Ltd., Tokyo, Japan).

### 2.4. FT-IR Assay

Fresh mycelia were lyophilized and ground with KBr (1:100). Then, the mixture was pressed into tablets for the test. Whole spectra in the wavelength range of 400–4000 cm^−1^ were recorded in the reflectance mode using an FT-IR spectrophotometer (ALPHA, Bruker Corp., Karlsruhe, Germany). Pure KBr was used as the background.

### 2.5. XPS Assay

The surface atomic changes of each specimen were characterized by a K-Alpha XPS system (K-Alpha^+^, Thermo Fisher Scientific, East Grinstead, East Sussex County, UK). Peak decomposition and data analysis were conducted using XPS peak 4.0 software. The test parameters were set as follows: energy: 1486.8 ev, test spot area: 400 μm, tube voltage: 15 kV, tube current: 10 mA, background vacuum: 2 × 10^−9^ mbar, and binding energy: 0–1400 eV. The survey scans were collected for binding energy spanning from 1100 eV to 0 with an analyzer pass energy of 50 eV and a step of 1.00 eV.

### 2.6. Proteomic Analysis of Cell Surface Proteins

Surface proteins of *A. flavus* were obtained according to the method reported previously with slight modifications [21]. In brief, 1 g of fresh mycelia were washed several times with deionized water, suspended in 5 mL of 0.156 M phosphate-buffered saline (PBS), and shaken for 3 h at room temperature. Supernatants were collected by centrifugation at 5000× *g* for 30 min and clarified by filtration with Whatman no. 2 paper and another centrifugation at 12,000× *g* for 3 min at 10 °C. Then, the content of surface protein in the collected supernatants was quantified by the Bradford assay [22]. Then, the proteins were separated by electrophoresis and trypsin hydrolysis. After desalination and lyophilization, samples were ready for analysis. Liquid chromatography-tandem mass spectrometry (LC-MS/MS) identification and Gene Ontology (GO) analysis, eukaryotic orthologous groups (KOG) functional classification, and Kyoto Encyclopedia of Genes and Genomes (KEGG) pathway analysis were conducted by the Beijing Genomics Institute (BGI, Shenzhen, China). In brief, proteins were separated through LC (eksigent ultra 2D, SCIEX, Framingham, MA, USA) and detected by Triple TOF 5600 (SCIEX, Framingham, MA, USA). The MS/MS data were aligned with *A. flavus* 3357 proteome (NCBI database).

### 2.7. Determination of the Activity of β-1,3-Glucan Synthase

Microsomal membrane preparation was conducted as previously described [23] except that fresh mycelia were ground with liquid nitrogen. Then, the activity of β-1,3-glucan synthase was measured as described elsewhere [24].

### 2.8. Determination of the Activity of Chitin Synthase

Prior to the determination of the activity of chitin synthase, the microsomal membrane was prepared and the microtiter plate was coated as described previously [25] except for the initial ratio of fresh mycelia and TM buffer containing Tris-HCl and MgCl_2_. In the present study, 12 mg of grounded fresh mycelia were resuspended in 10 mL of TM buffer. Then, chitin synthase activity was measured as described by Belewa et al. [24].

### 2.9. Determination of the Activities of β-1,3-Glucanase and Chitinase

Fresh mycelia were ground with liquid nitrogen and homogenized with extraction buffer (0.1 M sodium acetate buffer, pH 4.8) at a ratio of 1:2. Then, the homogenate was centrifuged at 10,000× *g* for 5 min at 4 °C. The supernatant was collected as crude enzyme extract. The activities of β-1,3-glucanase and chitinase were determined as described previously [26].

### 2.10. Real-Time Quantitative Reverse Transcription PCR (QRT-PCR)

The extraction of total RNA, reverse transcription, and real-time quantitative PCR were conducted with RC401 kit, P212 kit, and Q711 kit, respectively, following the instructions provided by the manufacturer (Vazyme Biotech Co., Ltd., Nanjing, China). The primers were synthesized by Sangon Biotech Co. Ltd. (Shanghai, China) and the sequences are shown in Table S1. A relative quantification method (2^−∆∆CT^) was used to evaluate changes in expression among three replicates.

### 2.11. Antifungal Effect of Paeonol on Peanut Butter

Peanut butter (Shandong Yinger Foodstuffs Co., Ltd., Zaozhuang, China) in a sealed can was bought from a local supermarket. Two grams of peanut butter were spread on a Petridish (d = 3.5 cm) and irradiated under UV light for 30 min. Then, fresh spores (2 × 10^5^ spores/Petridish) were mixed with paeonol at final concentrations of 0, 1/4 MIC, 1/2 MIC, and MIC. The growth of *A. flavus* was visualized every 24 h.

### 2.12. Statistical Analysis

All experiments were performed at least three times in triplicate. The results were presented as the mean ± SD. Significant differences were determined by one-way ANOVA using Duncan’s multiple range test for enzyme activities determination and Student’s *t*-test was used for QRT-PCR test (*p* < 0.05). The statistical analyses were performed by SPSS 20.0 (IBM, Armonk, NY, USA).

## 3. Results and Discussion

### 3.1. Paeonol Destroyed Both the Outer and Inner Layer of Cell Walls

**Figure 1:**
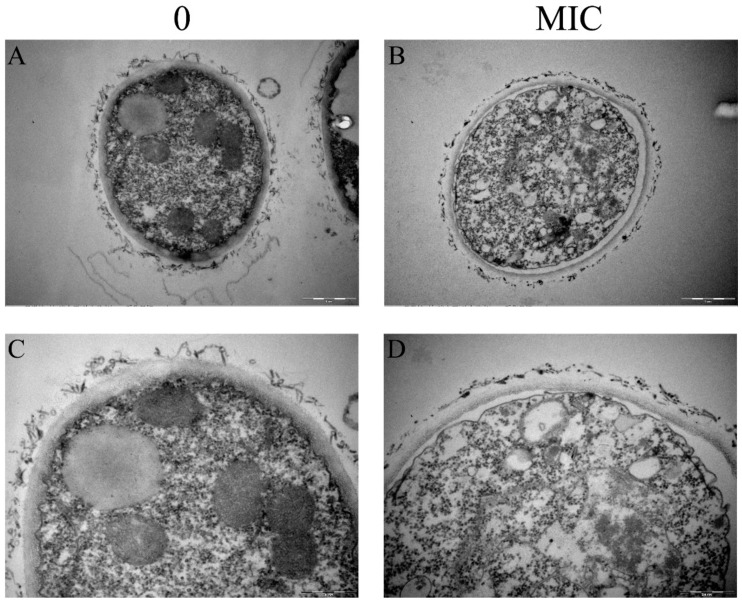
TEM characterization of *A. flavus* treated with paeonol at 0 (**A**,**C**) and MIC (**B**,**D**). Scale bar: 1 μm.

To understand the influence of paeonol on the ultrastructure of *A. flavus* mycelia, we conducted TEM characterization. As shown in Figure 1A,C, the cell walls of mycelia in the control group were clearly present with two layers, of which the outer layer is amorphous and the inner layer is fibrous. After paeonol treatment, both layers were thinner (Figure 1B,D). Quantification showed that the thicknesses of the inner layer were 199.17 ± 20.98 and 136.55 ± 10.92 nm for the control and the MIC treated group, respectively. As we determined previously, this reduction is attributed to the reduced content of β-1,3-glucan and chitin [17]. Therefore, paeonol treatment greatly damaged both the outer and the inner layer of *A. flavus* cell walls. Moreover, in the intact mycelia, some organelles such as mitochondria and vacuole are normal, while in the paeonol treated mycelia, the cytoplasm is in chaos. Plasmolysis can also be visualized in the paeonol treated mycelia. A similar mode of action was reported previously in *o*-vanillin treated mycelia, of which the chitin content was not changed remarkably [9].

### 3.2. Paeonol Changed OH Groups, Released Surface Proteins, and Increased Lipid Content

**Figure 2:**
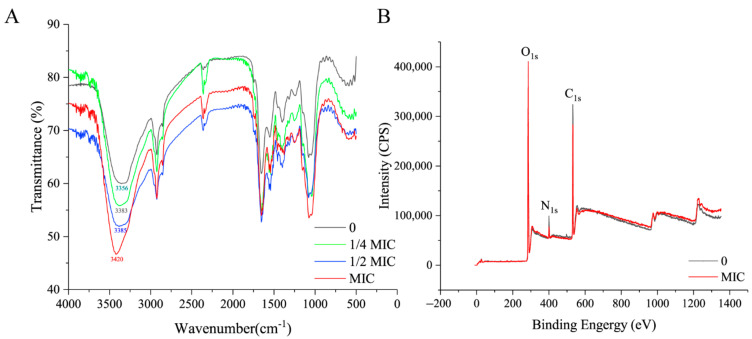
Influence of paeonol on the cell walls of *A. flavus*. (**A**) FT-IR spectra of mycelia treated with paeonol at 0, 1/4 MIC, 1/2 MIC, and MIC. (**B**) XPS survey spectra of mycelia treated with paeonol at 0 and MIC.

To determine the changes of functional groups in the surface of mycelia treated with paeonol, FT-IR characterization was conducted. As shown in Figure 2A, the peak around 3400 cm^−1^ (1) represents the O-H stretching vibration. Symmetric stretching vibration of CH_3_ group is present at 2925 cm^−1^ (2) [27,28]. The absorption band at 2360 cm^−1^ (3) was associated with stretching vibration of triple bond and cumulative double bond such as (-N=C=O, -N=C=O, etc.) [9]. The band at 1653 cm^−1^ (4) corresponds to the amide I band and amide II band, which are the characteristic infrared spectra of proteins [29]. The peak at the wavenumber of 1076 cm^−1^ (5) represents a symmetrical stretching vibration of C-O-C [30]. It can be observed that in the control group, the peak occurs at 3356 cm^−1^, and with the increase in the concentration of paeonol, the peak moved to higher wavenumbers, and at the highest concentration of paeonol (MIC), the wavenumber reached up to 3420 cm^−1^. For the other functional groups, no obvious changes can be detected. Therefore, paeonol altered the OH groups in the surface of *A. flavus* mycelia. Interestingly, *o*-vanillin also altered this group [9]. This is the first proof that, although belonging to different categories of plant-derived natural compounds based on chemical structure, paeonol and *o*-vanillin both altered OH groups.

The fact that OH groups are present in all three major compounds (polysaccharides, lipids, and proteins) in cell walls directed to further analysis. XPS is capable to determine the ratios of elements. Representative XPS survey spectra of *A. flavus* mycelia treated with paeonol at MIC and without are shown in Figure 2B. C, N, and O atoms occurred at about 532 eV, 400 eV, and 285 eV, respectively. The C_1s_ peak is composed of three components containing carbon in C-(C, H) at 284.4 eV, C-(O, N) at 286.3 eV, and C=O bonds at 288.0 eV. There are two components: O=C bonds at 531.4 eV and O-C bonds at 532.7 eV in the O_1s_ peak. The N_1s_ peak is also composed of two components: amine or amide functionalities at 399.9 eV and protonated nitrogen at 401.4 eV (Supplementary Figure S1). Among the three elements, only the nitrogen intensity was remarkably decreased from 8266.67 (the control group) to 6085.63 (the MIC group) (Supplementary Figure S1B,E). The quantification result in Table 1 showed that the content of C increased slightly while the content of N and O decreased. C is present in polysaccharides (Ps), lipids (Lp), and proteins (Pr). We adopted the method reported by Dague et al. [31] and determined changes of the three main components in the mycelia treated with paeonol. It showed that the content of polysaccharides was almost stable, however, a decrease (8.45%) of the content of protein and an increase (13.66%) of lipids occurred. Based on the altered OH groups, it is probably that this alteration decreased the proteins’ potential for hydrogen bonding. Consequently, paeonol could effectively release some proteins from the cell wall of *A. flavus* mycelia, which was similar to another plant-derived natural compound (*o*-vanillin) studied in our lab previously [9]. However, to the best of our knowledge, the composition of the surface protein is still unknown.

### 3.3. Identification of the Composition of Surface Proteins

**Figure 3:**
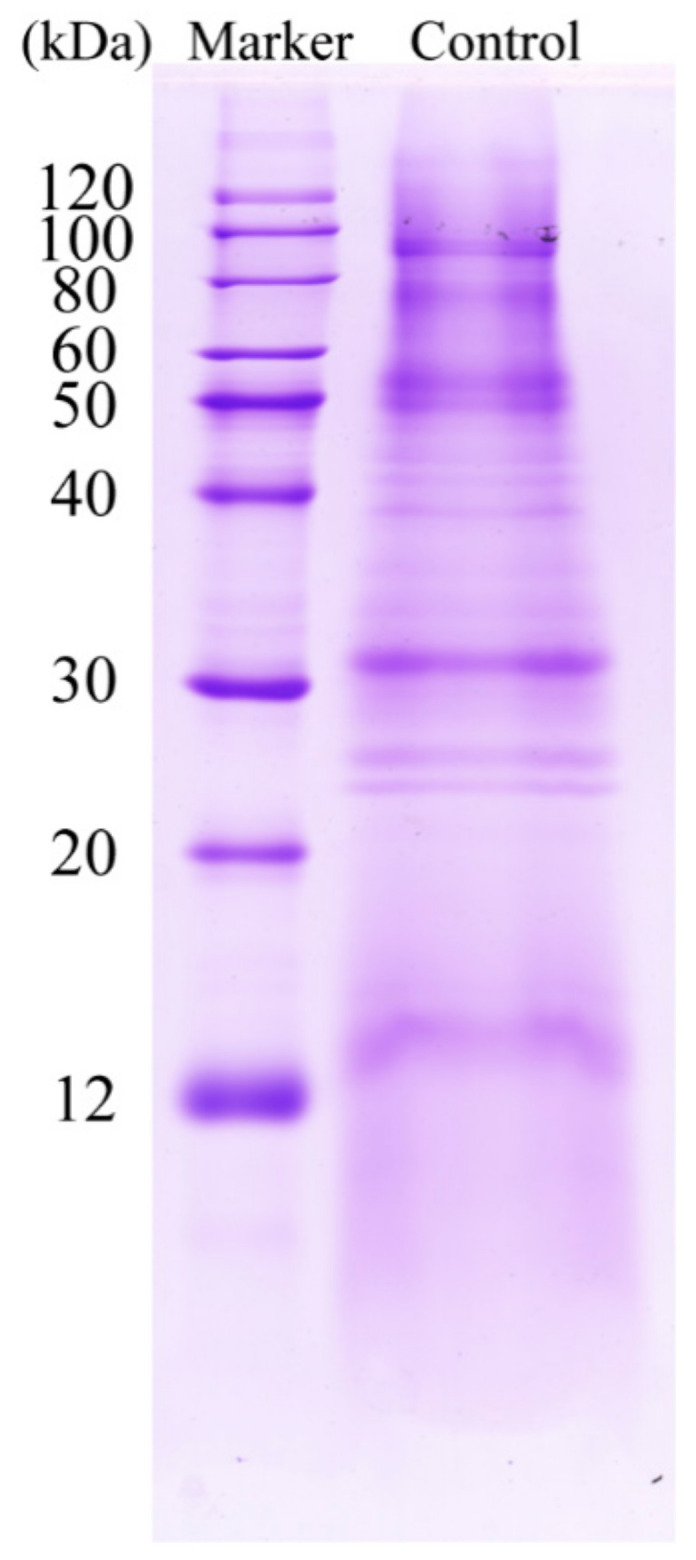
SDS-PAGE electrophoresis of *A. flavus* surface proteins.

Although a previous study reported that a varied number of proteins in the surface of *A. flavus* mycelia were present based on different detection methods [21], the detailed information of these proteins are still lacking, therefore, we conducted a proteomic analysis to identify them. As the integrities of cell walls and cell membranes are injured by paeonol treatment, intracellular components would be inevitably released during the collection procedure, confounding the components of surface proteins in paeonol treated mycelia. Therefore, only the surface proteins of intact mycelia (control group) were analyzed. The concentration of extracted surface proteins was 0.5149 ± 0.02 mg/mL according to the Bradford method. Before identification, SDS-PAGE electrophoresis was conducted, and as shown in Figure 3, a large number of proteins falling in a wide molecular range can be visualized, and among them, intensive proteins are around 90, 55, and 30 kDa.

**Figure 4:**
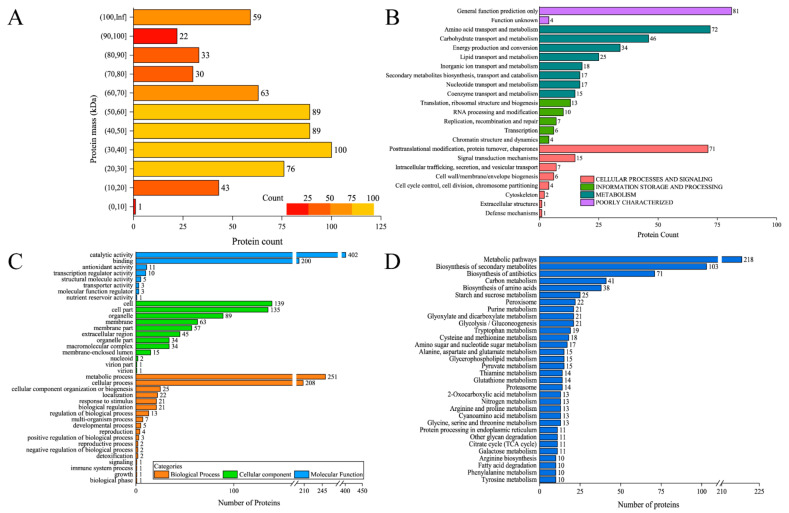
Identification and annotation of *A. flavus* surface proteins. (**A**) Protein count. (**B**) KOG analysis. (**C**) GO analysis (**D**) KEGG annotation.

By proteomic analysis, in total 605 proteins and 3639 peptides have been identified in intact mycelia. After alignment, all of the proteins have been identified. Although they are uncharacterized, their functions can be annotated by the GO, KOG, and KEGG pathways. First of all, the distribution of the proteins based on their mass is shown in Figure 4A. One hundred proteins are falling in the mass range of 30–40 kDa, and 89 proteins are in the mass range of 40–50 and 50–60 kDa, respectively. More than 50 proteins are falling in the range of 20–30, 60–70, and higher than 100 kDa. For KOG analysis, as shown in Figure 4B, there are 72 proteins classified in “amino acid transport and metabolism” and 71 proteins in “posttranslational modification, protein turnover, chaperones”. In Figure 4C, there are 590, 615, and 635 proteins classified in biological process, cellular component, and molecular function. To decipher the pathways the proteins participated in, we adopted KEGG analysis. According to the number of proteins in all categories, most of the proteins participated in the catalytic activity or metabolic process as these two biological functions rank the first in their respective category. As shown in Figure 4D, about 44% of proteins have been annotated in metabolic pathways, followed by biosynthesis of secondary metabolites and biosynthesis and antibiotics. These results indicated that *A. flavus* surface proteins are indeed involved in a myriad of biological functions.

Besides, we also annotated several interesting proteins involved in cell wall construction. As shown in Table 2, the proteins (XP_041141235.1, XP_041142920.1, XP_041147528.1) have been annotated as mannosyl-oligosaccharide α-1,2-mannosidase for the biosynthesis of GlcNAc, a precursor of N-glycan, in “various types of N-glycan biosynthesis”. The proteins (XP_041142920.1, XP_041146481.1, XP_041146483.1) have been annotated as mannosyl-oligosaccharide α-1,3-mannosidase and mannosyl-oligosaccharide α-1,2-mannosidase for N-glycan precursor trimming and biosynthesis. The proteins (XP_041141235.1, XP_041147528.1, XP_041151178.1) have been annotated as N-sulfoglucosamine sulfohydrolase and hexosaminidase in glycosaminoglycan degradation. The protein (XP_041148932.1) has been annotated as phosphatidylinositol N-acetylglucosaminyltransferase in glycosylphosphatidylinositol (GPI)-anchor biosynthesis. Notably, three proteins (XP_041143468.1, XP_041147095.1, XP_041147899.1) have been annotated as versiconal hemiacetal acetate reductase in aflatoxin biosynthesis. They have been indicated to mediate the conversion between 1′-hydroxy-versicolorone and versicolorone, versiconal-hemiacetal acetate and versiconol acetate, as well as versiconal and versiconol. Although the functions of these proteins have been not revealed, this is the first report of the identification and annotation of them (also their sequences), as well, the direct link between surface proteins and their roles in biological processes, in particular cell wall construction and aflatoxin biosynthesis.

### 3.4. Paeonol Regulated the Activities of Enzymes Participating in β-1,3-Glucan and Chitin Metabolism

The decreased content of both β-1,3-glucan and chitin by peaonol treatment directed the hypothesis that the metabolism of them were regulated. In a variety of yeasts and filamentous fungi, the synthases catalyzing the biosynthesis of polysaccharides in cell walls are located across plasma membranes [32,33]. After the linear polysaccharide chains are formed, they have to be remodeled and cross-linked to construct a 3D structural network during the neosynthesized polysaccharides by the enzymes (e.g., hydrolases and glycosyltransferases) [33,34,35]. Excessive polysaccharides are degraded by enzymes, such as glucanase, chitinase, etc., [32,33,36].

**Figure 5:**
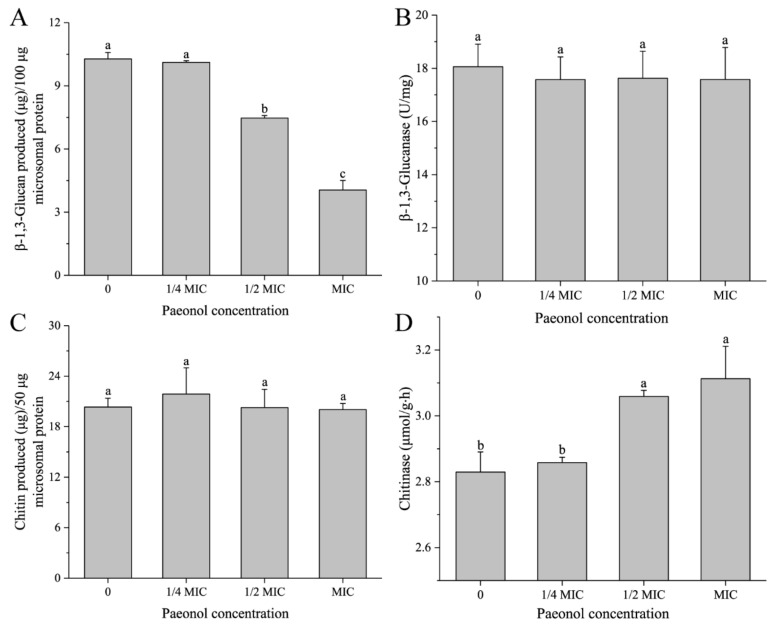
Activities of enzymes in the metabolisms of β-1,3-glucan and chitin in *A. flavus* treated with paeonol at 0, 1/4 MIC, 1/2 MIC, and MIC. (**A**) Activity of β-1,3-glucan synthase. (**B**) Activity of β-1,3-glucanase. (**C**) Activity of chitin synthase. (**D**) Activity of chitinase. a–c significant difference (*p* < 0.05) according to Duncan’s multiple range test.

To further understand the mode of action of paeonol on β-1,3-glucan and chitin in the inner layer of the cell wall, we quantified the amounts of β-1,3-glucan and chitin synthesized in vitro by β-1,3-glucan synthase and chitin synthase with nonradioactive methods, respectively, which can directly reflect the activities of β-1,3-glucanase and chitinase. As shown in Figure 5, except for the 1/4 MIC group, the content of neosynthesized β-1,3-glucan was significantly (*p* < 0.05) reduced by paeonol treatment in a dose-dependent manner (Figure 5A), indicating that the activity of β-1,3-glucan synthase was reduced. However, the activity of β-1,3-glucanase was relatively stable (Figure 5B). For chitin, the activity of chitin synthase was almost not changed as there were no significant (*p* > 0.05) differences in chitin production among the control and paeonol treatment groups (Figure 5C), and interestingly, in comparison with the control group, the activity of chitinase was promoted remarkably in mycelia with the exposure of paeonol at 1/2 MIC and MIC (Figure 5D). Therefore, we assume that paeonol could hinder the biosynthesis of β-1,3-glucan rather than enhance its degradation, as well, paeonol could promote the degradation of chitin rather than attenuate its biosynthesis. Our results are not fully consistent with previous reports. Belewa et al. claimed that extract from *Tulbaghia violacea* Harv. plant reduced the activities of both β-glucan synthase and chitin synthase, however, they did not determine the activities of glucanase and chitinase [24].

As β-1,3-glucan synthase and chitin synthase are inherent components of plasma membranes, their attenuated activities can also aggravate cell membrane damage. From this point of view, apart from the relative spatial position of cell walls and cell membranes, their functions are closely correlated with each other, providing some clues for further research in antifungal mechanisms.

### 3.5. Paeonol Regulated the Relative Expressions of Genes Encoding the Enzymes Participating in Β-1,3-Glucan and Chitin Metabolism

**Figure 6:**
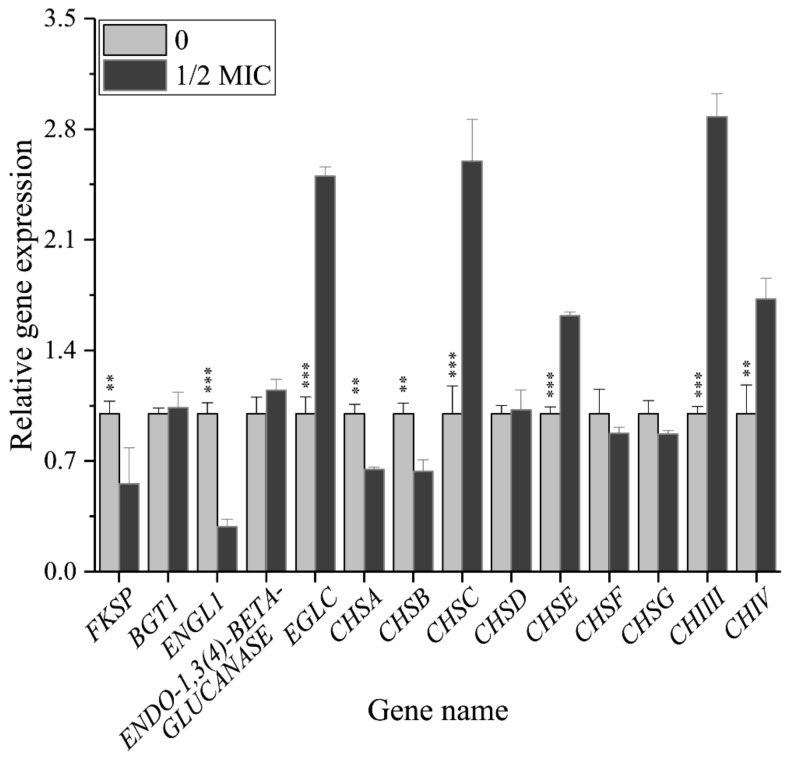
Relative gene expression of *A. flavus* treated with paeonol at 0 and 1/2 MIC. Student’s *t*-test, **: *p* < 0.01, ***: *p* < 0.001.

The above changes of activities of enzymes catalyzing β-1,3-glucan and chitin metabolism are indeed the comprehensive results of the expressions of several relevant genes participating in diverse functions, such as biosynthesis, reconstruction, and degradation. Therefore, quantifying changes of their relative expressions is of significance in revealing the antifungal effect of paeonol against *A. flavus* on the molecular level. Relative expressions of the genes are shown in Figure 6. *A. flavus* genome encodes only one β-1,3-glucan synthase, FKSP, and as expected, the relative expression of the *FKSP* gene decreased significantly with the treatment of paeonol at 1/2 MIC. We also quantified the expression level of the *BGT1* gene, which is responsible for the elongation of the β-(1,3)-glucans [33], it was not changed by paeonol treatment, therefore, paeonol reduced the biosynthesis of β-1,3-glucan only by downregulating the expression of *FKSP* gene. In the degradation of *A. flavus* β-1,3-glucan, there exist three key genes: *ENGL1*, *ENDO-1,3(4)-β-GLUCANASE*, and *EGLC*. Among them, the relative expression of *ENDO-1,3(4)-β-GLUCANASE* gene was not changed remarkably, while *ENGL1* gene and *EGLC* gene were downregulated by 0.71 fold and upregulated by 1.50 fold, respectively, probably leading to the unchanged activity of β-1,3-glucanase.

For chitin, in *A. flavus*, there are 7 genes mediating its biosynthesis: chitin synthase A, B, C, D, E, F, and G (*CHSA*, *B*, *C*, *D*, *E*, *F*, and *G*). Among them, the relative expressions of *CHSC* and *CHSE* were significantly upregulated, while *CHSA* and *CHSB* were remarkably downregulated by peaonol treatment, and *CHSD* and *CHSF* were not changed, probably resulting in an unchanged activity of chitin synthase. Although the impacts of *CHSA*, *B*, and *C* on chitin synthesis were limited in *A. fumigatus* [37], their actual functions in *A. flavus* have not been clarified. There are two classes of chitinase which are responsible for the degradation of chitin: class III (*CHIIII*) and class V (*CHIV*), the gene expressions of which were all upregulated remarkably by paeonol treatment, leading to the increased activity of chitinase. Therefore, we hypothesize that *FKSP*, *class III*, and *class V* genes might be the targets of paeonol in inhibiting the cell wall integrity of *A. flavus*. Although previous studies also reported plant-derived natural compounds in damaging cell wall integrity in conditional pathogens, the detailed mechanisms are not consistent. Hu et al. reported that *Perilla frutescens* essential oil downregulated α-1,3-glucan synthase *AGS1* gene and four chitinase genes in *A. flavus* [38]. Cinnamaldehyde was capable to target β-1,3-glucan synthase and chitin synthase in *Saccharomyces. Cerevisiae* [39], as well as regulate glucanase, chitin synthase, and chitinase in *Geotrichum citri-aurantii* [15].

### 3.6. Paeonol Can Effectively Control the Pathogenicity of A. flavus on Peanut Butter

**Figure 7:**
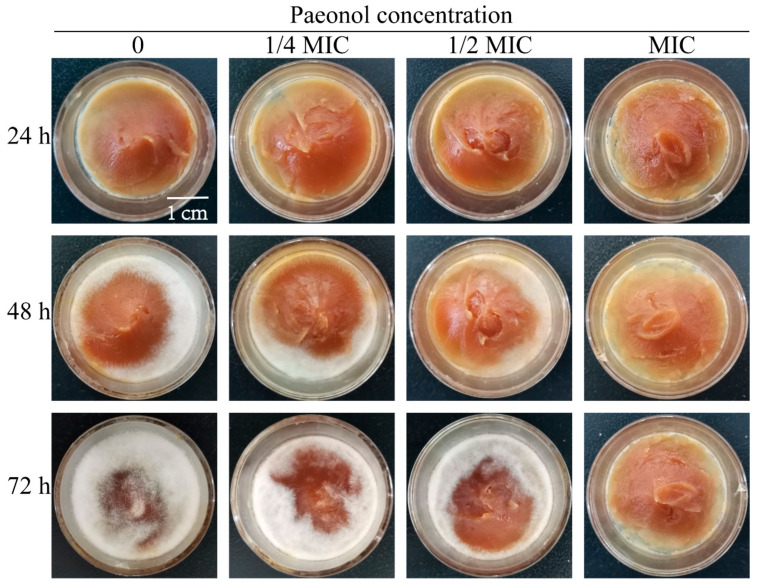
Antifungal effect of paeonol in vitro on peanut butter.

In our previous study, paeonol performed a strong inhibitory effect on peanuts and corn kernels [17]. In the present work, we continued the determination of agricultural products. Peanut butter was selected to evaluate the antifungal effect of paeonol. As shown in Figure 7, white mycelia can be visualized on peanut butter at 48 h for those not treated with paeonol, and almost the whole surface of peanut butter was covered with *A. flavus* for 72 h. For those treated with paeonol, the growth of *A. flavus* was restrained in a dose-dependent manner. In comparison with the control, fewer mycelia were covered on the surface of peanut butter treated with paeonol at 1/2 MIC for 72 h. Paeonol at MIC can fully suppress the growth of *A. flavus* in the tested period. Therefore, paeonol can also effectively control the contamination of *A. flavus* on peanut butter.

## 4. Conclusions

Paeonol can destroy both the outer and inner layers of *A. flavus* cell walls. The proteins identified in the outer layer were involved in many biological processes, interestingly, several of them participated in the construction of the inner layer as well as aflatoxin biosynthesis. The biosynthesis of β-1,3-glucan was damaged by paeonol treatment via attenuating β-1,3-glucan synthase encoded by *FKSP* gene, while the degradation of it was not changed. The activity of chitinase was reduced and it was mainly due to the downregulated expressions of *class III* and *class V chitinase* genes. In addition, paeonol performed strong inhibitory efficacy against *A. flauvs* on peanut butter. This study revealed a new mode of action of paeonol damaging cell walls of *A. flavus*, as well as providing a theoretical basis for the application of paeonol in the preservation of agricultural products.

## Figures and Tables

**Table 1:** Surface chemical composition measured by XPS of mycelia treated with paeonol at 0 and MIC.

	%C	%N	%O	N/C	O/C	N/O	%C_Pr_	%C_Ps_	%C_Lp_
0	72.57	5.57	21.86	0.08	0.30	0.25	19.96	18.45	34.15
MIC	77.72	3.21	19.07	0.04	0.25	0.17	11.51	18.40	47.81

**Table 2:** Proteins and the pathways involved in cell wall construction and aflatoxin biosynthesis.

Pathway	Protein Name
Other glycan degradation	XP_041140530.1;XP_041141235.1;XP_041141593.1;XP_041142346.1;XP_041144446.1;XP_041145896.1;XP_041147338.1;XP_041147528.1;XP_041148927.1;XP_041151327.1;XP_041151574.1
Various types of N-glycan biosynthesis	XP_041141235.1;XP_041142920.1;XP_041147528.1
N-Glycan biosynthesis	XP_041142920.1;XP_041146481.1;XP_041146483.1
Glycosaminoglycan degradation	XP_041141235.1;XP_041147528.1;XP_041151178.1
Aflatoxin biosynthesis	XP_041143468.1;XP_041147095.1;XP_041147899.1
Glycosylphosphatidylinositol (GPI)-anchor biosynthesis	XP_041148932.1

## Data Availability

Raw data can be provided by the corresponding author on request.

## Supplementary Materials

The following are available online at https://www.mdpi.com/article/10.3390/foods10122951/s1, Figure S1: XPS scan of C1s, N1s, O1s region of *A. flavus* surface treated with paeonol at 0 and MIC, Table S1: Primer sequences for QRT-PCR.
