# Supplementary material for: Paeonol Disrupts the Integrity of Aspergillus flavus Cell Walls via Releasing Surface Proteins, Inhibiting the Biosynthesis of β-1,3-Glucan and Promoting the Degradation of Chitin, and an Identification of Cell Surface Proteins
Source: Foods. 2021 Dec 1;10(12):2951. doi: 10.3390/foods10122951 (PMC8700735; doi:10.3390/foods10122951)
Supplement: Supplementary file 1 [file foods-10-02951-s001.zip › foods-1450799-supplementary.pdf]

Table S1. Primer sequences for QRT-PCR

Gene Name	Primer sequence (5′-3′ forward/reverse)
18S rRNA	CTGCGGAAGGATCATTACCGAGTG<br>GGACGACGACCCAACACACAAG
1,3-BETA-GLUCAN SYNTHASE CATALYTIC SUBUNIT FKSP	ATTGACGCCAACCAGGACAACTAC<br>TCACCAAGGACACCGACACTCTC
1,3- BETA-GLUCANOSYLTRANSFERASE (BGT1)	GTACTGCCGATCCCATCATCCAAG<br>CCTTAGCAAGCCTCGTGTCATTCC
ENDO-1,3-BETA-GLUCANASE ENGL1	TGCCATCTGTCACATTGCCTTCAC<br>AGATAGAGGTTGGAGCGGTGGTAG
ENDO-1,3(4)- BETA-GLUCANASE	ACCAACCAACGACAGCGACAAC<br>CGTAGTTCCACCGTCCGAAGAATC
GPI-ANCHORED CELL WALL BETA-1,3-ENDOGLUCANASE EGLC	GTCGGATGGCAGCGTCAAGTC<br>AGCGGGAATGGCGGAGATAGG
CHITIN SYNTHASE A (CHSA)	CACAATCTACATGGCAGTCGTCCTC<br>ACCACGGAACATTCGCACAACC
CHITIN SYNTHASE B (CHSB)	CGAACATTCTGGACAAGCCTCTGG<br>GCCGTGAACACATCCGCATCC
CHITIN SYNTHASE C (CHSC)	TCTTCCTATTAGCCTCGTCGTCCTC<br>GGTATGGTCGGTTCGTTCGTTCC
CHITIN SYNTHASE D (CHSD)	CTTCCTCCACTGGTTCGCATTCTG<br>CGGTCGTCCAAGACTTCCTTTATCC
CHITIN SYNTHASE E (CHSE)	ATCTTCATCTTCCGCCGCAAGTG<br>GTCATCCATGTGCCAGAACGAGTAG
CHITIN SYNTHASE F (CHSF)	AGACCATCCCAACTACCACTACCG<br>GCCTATCCATGCTGCGAGAACTG
CHITIN SYNTHASE G (CHSG)	GACACCGAACCAGCAACTCATCC<br>ACGACCGAAGGCATTGAACAGC
CLASS III CHITINASE (CHIIII)	ACACTGGCATCACGCACATCATC<br>AGCGGCTCCTCCTAGCATTCC
CLASS V CHITINASE (CHIV)	TGGTGGTATGTGGTGGGAGACTAG<br>GGCTCGCAGGTTGTCGTACTTG

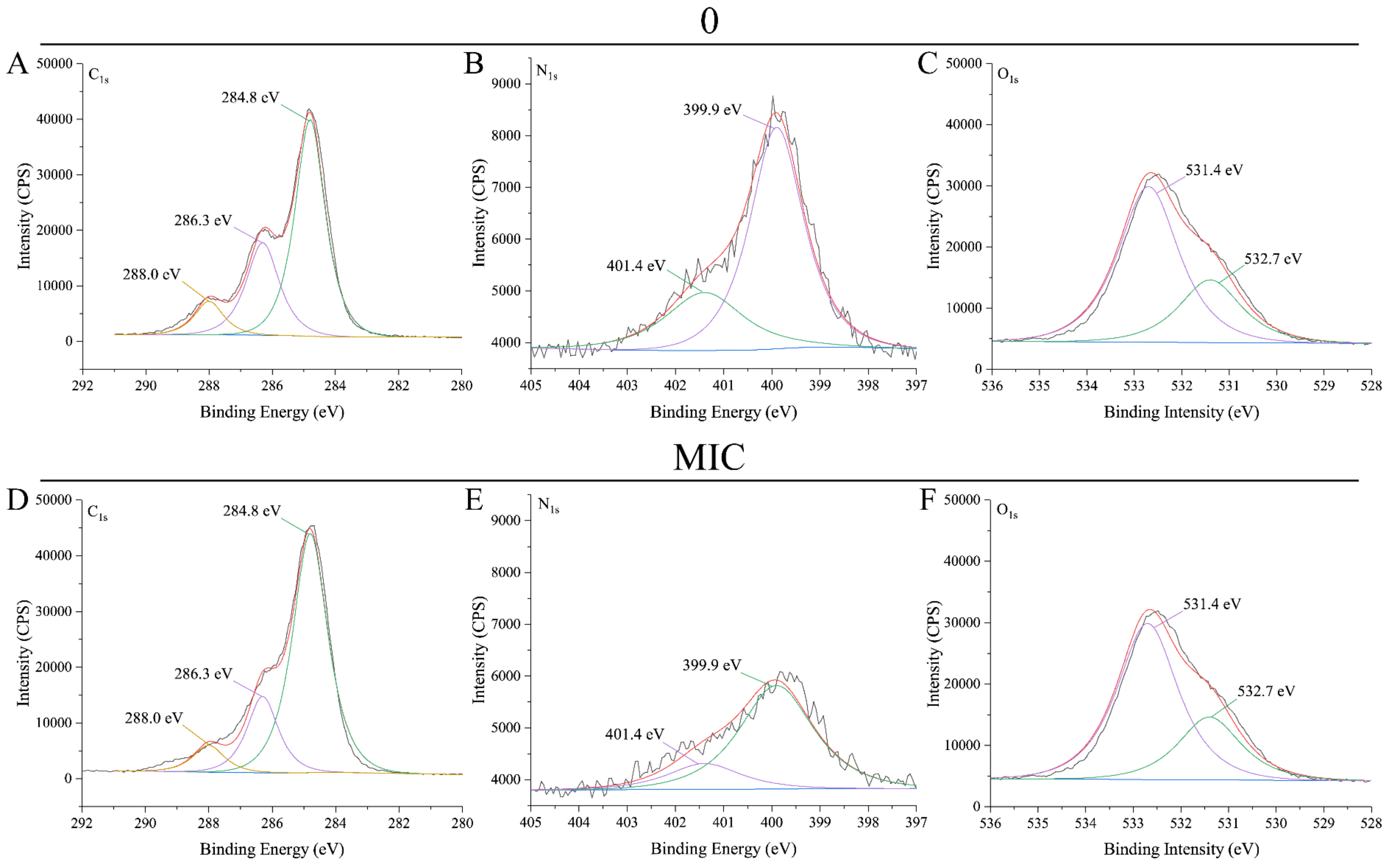

Figure S1: XPS scan of C1s, N1s, O1s region of *A. flavus* surface treated with paeonol at 0 and MIC
